# Sedimentary conditions based on the vertical distribution of radionuclides in small dystrophic lakes: a case study of Toporowe Stawy Lakes (Tatra Mountains, Poland)

**DOI:** 10.1007/s11356-022-21922-3

**Published:** 2022-07-19

**Authors:** Katarzyna Szarłowicz, Marcin Stobiński, Filip Jedrzejek, Barbara Kubica

**Affiliations:** Faculty of Energy and Fuels, AGH University of Science and Technology, al. A. Mickiewicza 30, 30-059 Krakow, Poland

**Keywords:** Dystrophic lakes, Sediments, Natural radionuclide, ^137^Cs, ^241^Am, ^210^Pb-method, Sedimentary conditions

## Abstract

The aim of this work was to prove the use of radionuclides deposited in sediment core taken from an overgrowing dystrophic lakes surrounded by marsh-peat vegetation to estimate sedimentary conditions. Sediment core samples were taken from the Toporowe Stawy Lakes (Niżni (TSN) and Wyżni (TSW); Tatra Mountains). The sampling was done using a Limnos corer. After the physical sample preparations, gamma measurements were performed. Radiochemical analysis was applied with the aim of determining ^210^Pb radioactivity by means of ^210^Po. The mean values for TSN lake are as follows: ^137^Cs ~ 123 Bq∙kg^−1^, ^40^ K ~ 389 Bq∙kg^−1^, ^228^Th ~ 55 Bq∙kg^−1^, ^226^Ra ~ 86 Bq∙kg^−1^, ^241^Am ~ 5 Bq∙kg^−1^, and ^210^Pb_uns_ ~ 180 Bq∙kg^−1^. For TSW lake, the radioactivity levels of ^226^Ra and ^241^Am are comparable to the TSN. The mean values of ^137^Cs, ^40^ K, and ^228^Th are almost twice as high as in TSN. The level of ^210^Pb in uppermost layer of TSN is higher than in TSW. Sediments were dated by use of ^210^Pb method, and the rate of sedimentation of each layer was also estimated. Basic chemometric tools were used to confirm the way of deposition of radionuclide, find the correlations between variables, and compare analyzed lakes. It was concluded that the presented type of lakes are a valuable source of information and the vertical distribution of radionuclide can be used to interpret the source of material supply and factors that influence the sedimentation process in recent 150–200 years.

**Supplementary Information:**

The online version contains supplementary material available at 10.1007/s11356-022-21922-3.

## Introduction

The water ecosystem provides documents and evidence with high sensitivity in order to study global climate changes and sedimentary environment. Mountain lake sediments are a valuable research material because their accumulation occurred in places located relatively far from urban and industrial areas. Lake sediments can be used to observe environmental changes both on a regional and global scale (Hamerlík et al. 2016; Wang et al. 2018). Sediments constitute an important component of water ecosystems. The intensity of sedimentation, the thickness of the layer of sediments, and their granulometric and chemic composition depend on the geological structure of a given reception basin, the geomorphology of the terrain, morphometry (in the case of lakes), climatic conditions, and the whole range of processes which occur in the standing bodies of water themselves (Charles et al. 1990; Du et al. 2021; Kanhaiya et al. 2018; Valette-Silver 1993). The accumulation of heavy metals, highly toxic organic substances, and other instances of pollution in sediments contributes, on the one hand, to the self-purification of the water environment, and, on the other hand, they are a constant source of the secondary pollution of standing bodies of water (Grba et al. 2017, Last and Smol 2001, Nõges et al. 1999, Sandor et al. 2001). The majority of substances in water ecosystems pass to sediments, and, as a result of this, they frequently contain high concentrations of pollution, and their concentration in water may be in an acceptable range of a norm.

Natural radioactivity in soils and rock sediments is mainly due to ^238^U and ^232^Th and primordial ^40^ K radionuclide. ^226^Ra belongs to uranium-radium decay series. Thorium is the most abundant natural radioactive element. The concentration varies considerably, depending on the geological characteristic of the area (Janković et al. 2008; Kritsananuwat et al. 2015; Mandujano-García et al. 2016; Shams et al. 2016; Skoko et al. 2021).

Additionally, human activity has caused an increase in radioactivity in the environment. ^134^Cs and ^137^Cs are major fission products in nuclear processes, and, with half-lives of 2.1 and 30.07 years, respectively, they constitute an important source of contamination of the environment with radioactivity (Baverstock and Williams 2007). Especially attention is given to ^137^Cs. It is a very important component of radioactive fallout; and because of its moderately long half-life and high solubility, it is a major source of long-lived external gamma radiation from fallout. ^137^Cs was introduced into the environment in huge amount (163 × 10^15^ Bq) during nuclear weapons tests (years 1945–1963) and was emitted into the atmosphere during the Chernobyl disaster (85 × 10^15^ Bq) (Lehto and Hou 2010). ^134^Cs was also introduced into the environment during the Chernobyl accident (46 × 10^15^ Bq ^134^Cs). Currently, its amount, among others in Poland, is not measurable due to the short half-life (CLOR 2021). However, during the nuclear accident in Fukushima, a new amount of radiocesium was distributed into the environment (^137^Cs, 13 PBq; ^134^Cs, 11.8 PBq). ^241^Am was also introduced to the environment during the mentioned accidents. It is the most important radionuclide of americium, with a half-life of 433 years. It will remain detectable in lake sediments for several centuries (Appleby et al. 1991). Most radionuclides are sorbed directly into sediments or suspended matter within 1–2 years (Bolsunovsky et al. 2005, Evangeliou et al. 2013, Hakonson and Whicker 1975). However, under unfavorable conditions, they can be remobilized from sediment causing water contamination (Davison et al. 1993; Szarlowicz et al. 2011).

Many studies focus on mountain lakes, but most of them refer to high mountain lakes. Particularly this is geomorphological, biological, paleobiological, chemical, etc. research. Some of these analyses allow one to recreate the environmental conditions that prevailed at a given time, as well as to determine the age of formation of lake sediments (Appleby and Piliposian 2006, Bitušík et al. 2018, Kuefner et al. 2020, Reczyński et al. 2020, Szarlowicz et al. 2018). Apart from the lakes, which are now fully developed, glacial lakes with swamp-peat sediments deserve attention. Examples of such lakes are found in the Tatra Mountains; they are located in the lower floors of the Tatra massif, mainly in the mountain range. There are casting cavities that are permanently or periodically filled with water, such as Toporowe Stawy Lakes (Toporowy Staw Niżni Lake (TSN) and Toporowy Staw Wyżni Lake (TSW)). From 2018, this area was included in the list of wetlands of international importance (the so-called Ramsar Convention).

The purpose of this study was to prove whether sediments collected from an overgrowing dystrophic lake surrounded by marsh-peat vegetation can be used to estimate sedimentary conditions and indicate source of materials supply based on radionuclide analysis, in the same way as is the case with other high-mountain lakes. To prove this, two lakes that were formed as a result of the same glacial processes lying very close to each other and showing mostly the same parameters were chosen. The specific goals of this work were to determine the radioactivity of chosen radionuclides in the sediment core to evaluate the level of the presence the radionuclide during sediment core and estimate the source/origin of them and check whether in selected lakes it is possible to use the determination of the age of individual layers using the ^210^Pb method, the estimation of the sedimentation rate, the use of statistical methods to differentiate the studied objects, and the indication of the correlation between the studied variables as well as confirmation of the sources of radionuclide supplies to the lakes.

## Materials and methodology

### Study area

The Tatra Mountains are a mountain range located on the Polish-Slovakian border. It forms the highest part of the Carpathian range and occupies an area of more than 785 km^2^. The peak heights are in the range between 900 and 2655 m above sea level with the highest peak being Gerlach. The Tatras are characterized by a mountain climate (extreme temperatures range, high moisture) and alpine scenery with a unique landscape. The Tatra National Park (both Polish and Slovakian) was created to protect the natural environment, and tourism has been limited. The mountains were formed during the alpine glaciation and have a varied geological structure. The structure is divided into three parts: the southern region (metamorphic rocks and granites), the middle region (carbonate rock), and the northern region (dolomite rock, slate) (Baumgart-Kotarba and Kotarba 2001, Klimaszewski 1988, Skiba 2007).

**Fig. 1:**
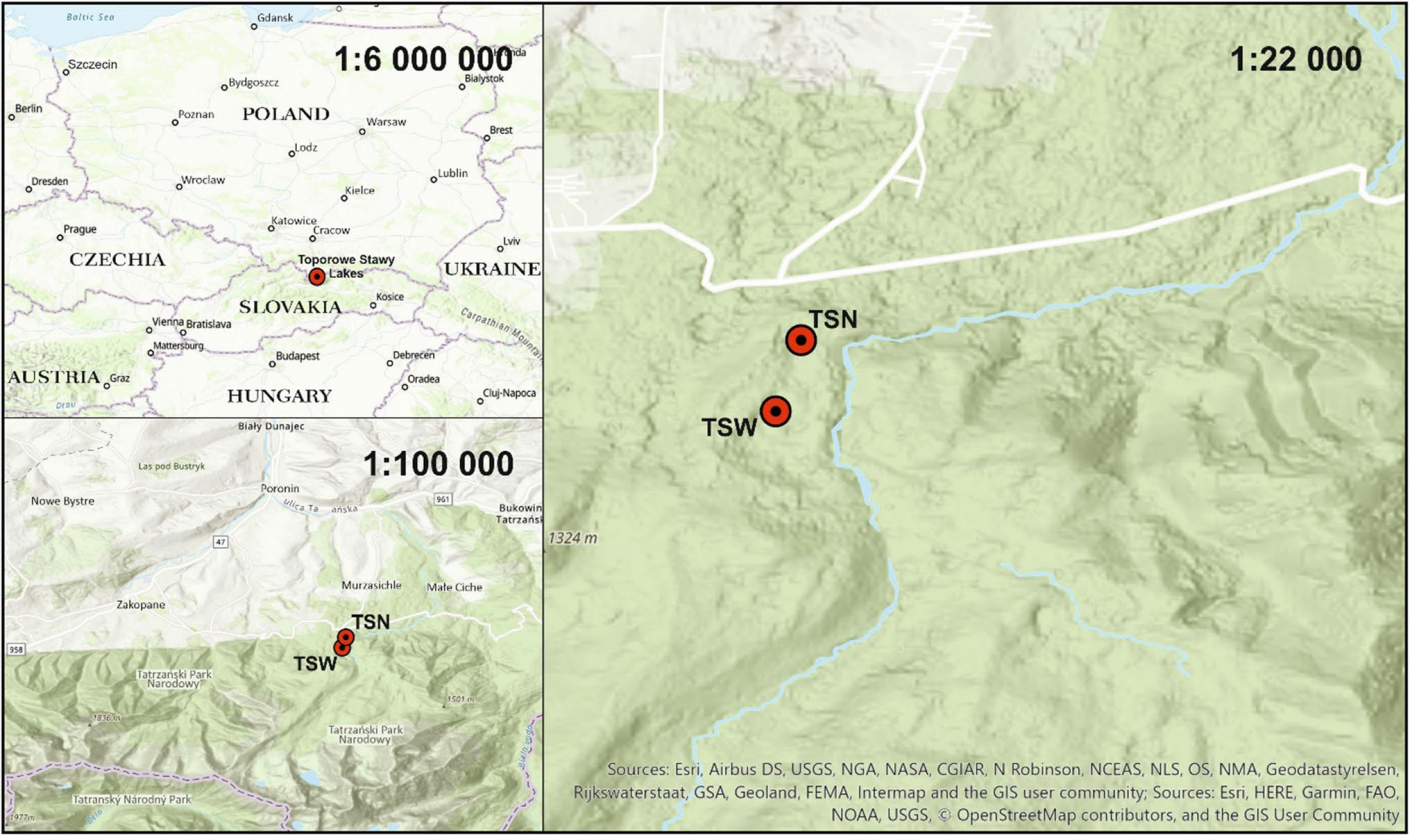
Study area—Toporowe Stawy Lakes (TSN, Toporowy Staw Niżni Lake; TSW, Toporowy Staw Wyżni Lake) (Tatra Mountains)

The research area is located in the southern region in the Sucha Woda Gasienicowa valley. It is rich in small-retention lakes. The Toporowe Stawy Lakes are the only lakes in the Tatra Mountains located in the lower regions of the region rock crests and frontal moraines formed by the merger of glaciers. It was then that the so-called the Moraine Amphitheater of Toporowe Stawy Lakes. The moraine amphitheater consists of three moraine embankments between which depressions occur with water, or peat inside (Mułenko et al. 2008) shows a map with the TSN and the TSW at a distance of 400 m to the south (Fig. 1). Table 1 provides basic information on these lakes (Bembówna 1969; Mirek 1996). According to the geological maps of the Polish Geological Institute, the lakes are located on the ground of shale (TSN) and dolomites (TSW). The catchment areas also include the nummulite limestone. The surface layer of the catchment area is made of irregular stone rubble, covered with peat in the basin of lakes (PGI 2015). The lakes are both endorheic, which favors the overgrowing of the lakes, and the catchment area is relatively small as a consequence of alpine-like terrain. The cumulated catchment area is 0.56 km^2^, and the size was obtained by analyzing a GIS topographic data. The average annual precipitation for the Tatra region is 1263 ± 206 mm (data gathered from Institute of Meteorology and Water Management). The TSW has a smaller area and is shallower, but both reservoirs are moraine-dammed lakes and therefore have quite similar characteristics.

**Table 1:** Basic information of Toporowe Stawy Lakes

Characteristic	Toporowy Staw Niżni Lake	Toporowy Staw Wyżni Lake
Area	0.617 ha	0.03 ha
Medium depth	1.9 m	1.1 m
Altitude	1089 ma.s.l	1120 ma.s.l
Summer temperature amplitudes	4–22 °C	4–22 °C
Flora	Peat-bog formation	Peat-bog formation
Conditions	Dystrophic lake	Dystrophic lake
Retention	Small	Very small

The location of the lakes (the strict protection area) limits direct human influence and is a representative research target of anthropopressure from indirect impact. The main sources of pollution are the surroundings of the lake and the so-called long-range impact. It is worth emphasizing that in the Tatra Mountains, a short industrial activity was recorded consisting in obtaining ores, mainly silver, copper, and iron. There was also a metallurgical work operating in the area from the beginning of the nineteenth century for about 70 years. The operation of these plants was short due to the low concentrations of ore and the difficult terrain conditions (Paryski and Radwanska-Paryska 2004). In addition, there was an episode of radiological significance in the history of the region. In the 1950s, an attempt was made to exploit uranium deposits in one of the Tatra valleys. Two mining adits were dug, but no longer exploitation took place due to the low concentration of uranium. However, it may impact the rate of radon exhalation (Kozak et al. 2013).

### Sampling and preparation for measurements

**Fig. 2:**
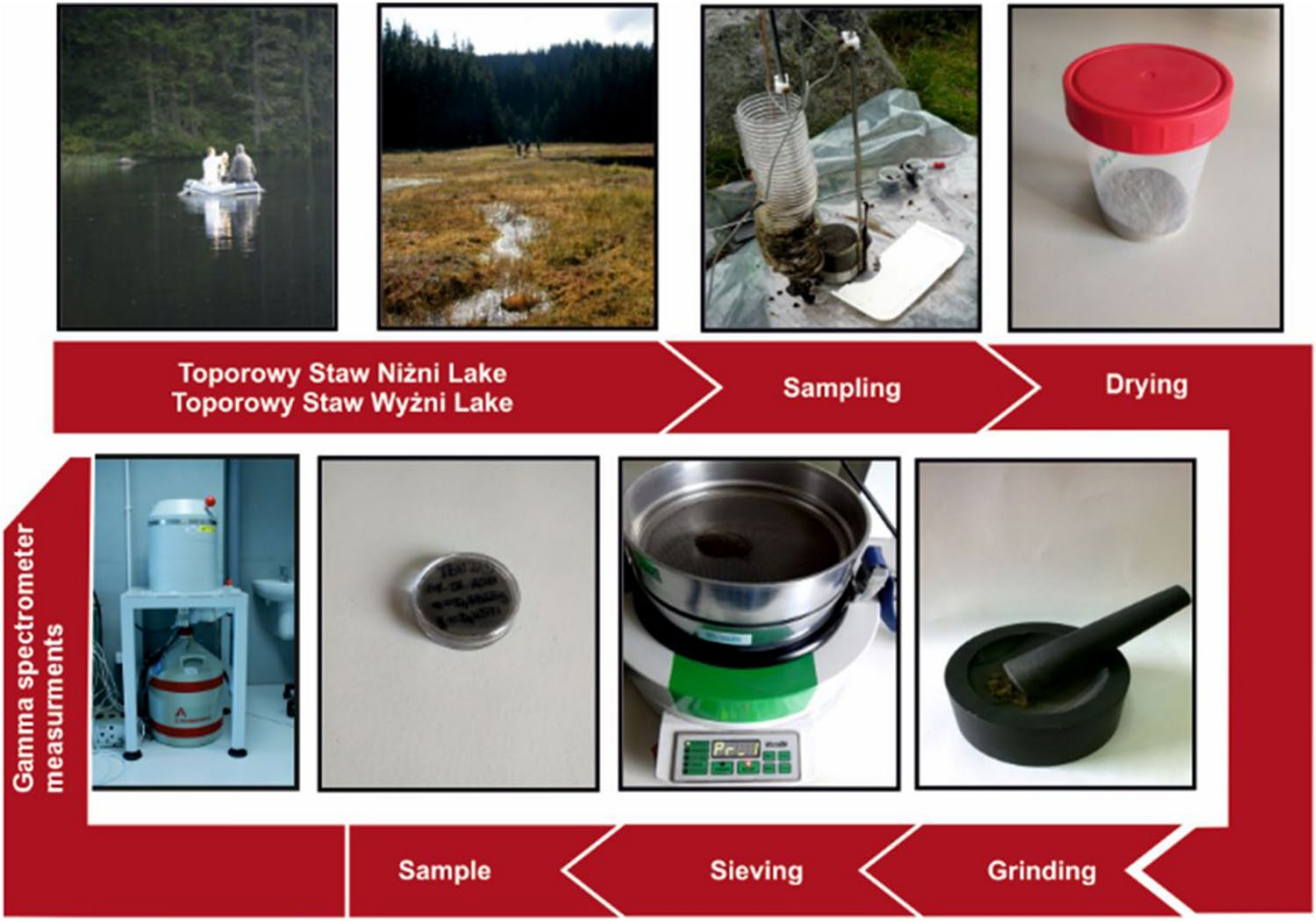
The analytical process in the determination of gamma radionuclides in sediments

Sediment samples were collected using a Limnos corer. The TSN samples were taken from the northern end of the lake at a distance of about 4 m from the rushes on the lake axis at a depth of about 3 m from a pontoon. Due to the fact that TSW is an overgrown lake, in order to collect the material, the collecting persons moved toward the lake from where the samples were taken. For TSW, the material was obtained from the lake on the eastern shore at a depth of approximately 1 m. The sediment core was divided into 1 cm layers and packed into containers. In the laboratory, the samples were air-dried, ground in a mortar, and sieved. Finally, dried homogenized samples were packed into a hermetically sealed measuring vessel (cylindrical shape, 1.5 cm^3^ volume (full-filled, mass of the sediments around 1 g), made of polystyrene). All procedure was presented in Fig. 2. In chosen samples, the organic matter content was estimated as loss on ignition (LOI). The results were expressed as the percentage weight loss after combustion at 550° C (Heiri et al. 2001).

### Gamma-ray measurements

The radioactivity of selected radionuclides in sediment samples was determined by gamma-ray spectrometry. The measurements used Broad Energy Germanium detector by Canberra Packard (BE 3830) of 34% relative efficiency. The detector was efficiently calibrated using three different reference materials: IAEA-447 (^137^Cs, ^241^Am, ^40^ K), IAEA-RGU-1 (uranium series), and IAEA-RGTh-1 (thorium series). Details on the energy of the gamma rays, which was used in determination of selected radionuclides, are shown in Table 2. Before analysis, containers were stored for at least 4 weeks to reach secular equilibrium between ^226^Ra and ^222^Rn. Finally, each sample was measured between 3 (at least) and 7 days. The measurement time was extended (more than 3 days) in order to achieve better counts statistics, taking into account also background counts statistics.

**Table 2:** Gamma energy lines for analyzed radionuclides

Radionuclide	Method
^137^Cs	Directly: 661.7 keV
^40^ K	Directly: 1460.8 keV
^241^Am	Directly: 59.5 keV
^226^Ra	Equilibrium assumption, mean value: 1764.5 keV, 1120.3 keV, 609.3 keV (^214^Bi) 351.9 keV, 295.2 keV (^214^Pb)
^228^Th	Equilibrium assumption, mean value: 2614.5 keV, 583.1 keV (^208^Tl)* 727.3 keV (^212^Bi) 238.6 keV (^212^Pb)

^*^Branching ratio of ^212^Bi (35.94%) was taken into account

The calculation of the radioactivity was based on the following formula.

$$A=\frac{{\mathrm N}_{\mathrm s}-{\mathrm N}_{\mathrm b}}{\mathrm m\bullet\mathrm T\bullet\mathrm p\left(\mathrm E\right)\bullet\varepsilon\left(\mathrm E\right)\bullet{\mathrm T}_{\mathrm z}}\left[\mathrm{Bq}\cdot\mathrm{kg}^{-1}\right],$$

where *N*_*s*_ is the number of sample counts, *N*_*b*_ is the number of background counts, *m* is the mass of the sample (kg), *T* is the measurement time, *p* (*E*) is the probability of emission (*E*)–peak efficiency, and *T*_*z*_ is the self-absorption coefficient.

The expanded uncertainty of the results included the following: the uncertainty of the radioactivity of the reference material, the uncertainty of the background measurement, the uncertainty of the efficiency measurement, and the standard efficiency for the sample measurement.

### Radiochemical analysis and alpha measurements

The radioactivity of ^210^Pb was determined via its daughter radionuclide ^210^Po. Polonium was deposited on a silver disc after the radiochemical analysis proposed by Szarlowicz et al. (2018). Two depositions were made in time of around 6 months; 0.1 g of dried sample with concentrated hydrochloric and nitric acid was digested in the Anton Paar microwave system. The samples were then centrifuged and evaporated with 2 mol dm^−1^ HCl; polonium was spontaneously deposited on a silver disc (Flynn 1968) in the presence of hydroxylamine hydrochloride or ascorbic acid within 3 h at temperatures of 85–88 °C. An alpha spectrometer (Alpha Analyst, Canberra, USA) with a PIPS (passivated implanted planar silicon) semiconductor detector was used for polonium measurements. The detector energy and efficiency calibration was performed using Standard Reference Source 99,981, source with a ^208^Po and AMR-33 source. The measurement time was 3 days for all sources. All spectra were analyzed using Genie-2000 software. The uncertainty was calculated using the total differential.

### Age determination (^210^Pb method) and sedimentation rate

**Fig. 3:**
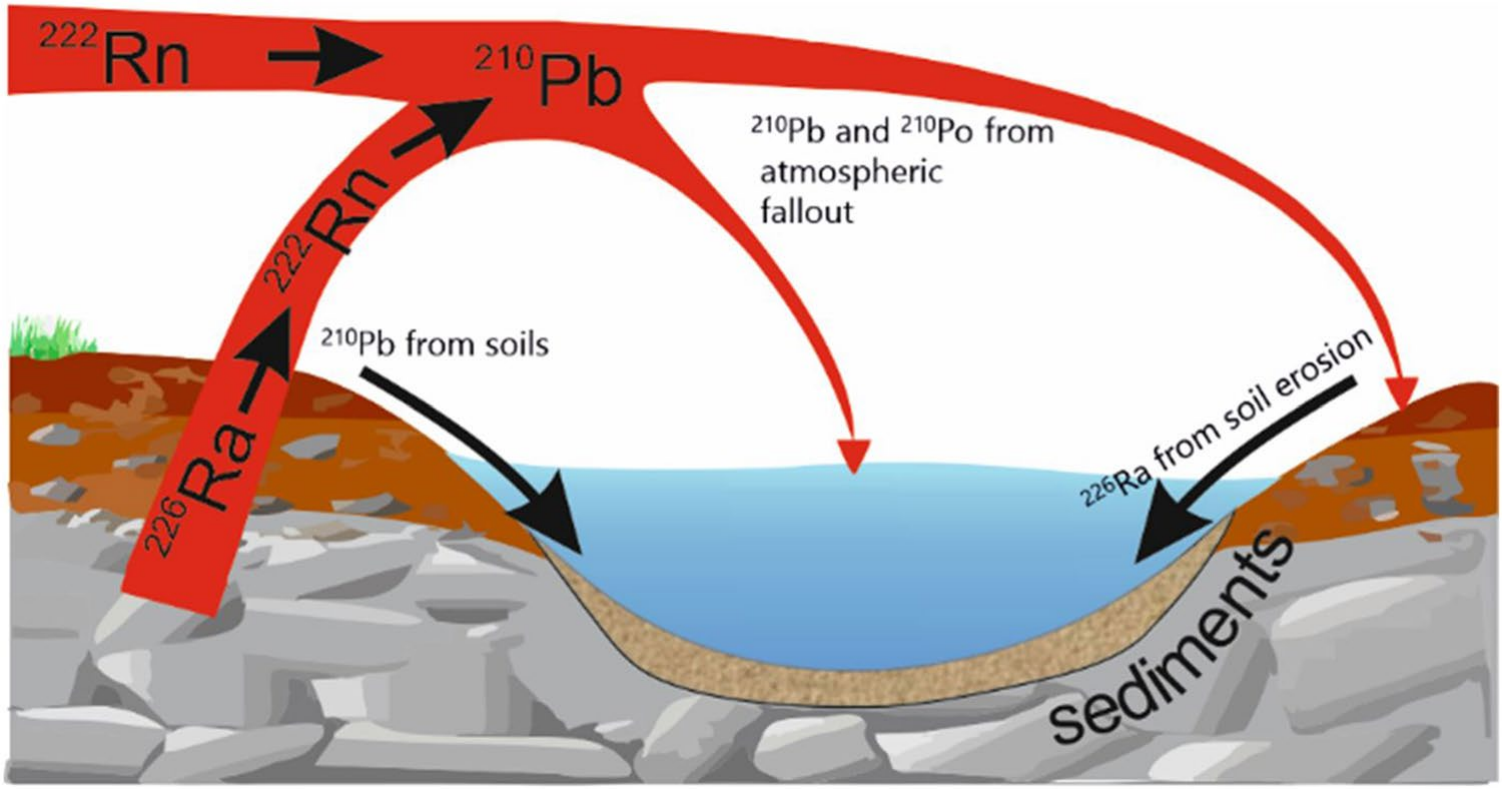
Sources of ^210^Pb in sediment

The calculation of the age of sediments is conducted on the basis of the law of radioactive decay and the changes of the concentration of the so-called unsupported lead by the application of an appropriate model. The most frequently used model is the CRS (*constant rate of supply*) model, which stipulates that the fallout of ^210^Pb_unsup_ from the atmosphere to water is constant (Appleby and Oldfield 1978). This component of lead is supplied to a standing body of water as a result of the decay of ^222^Rn which arose from the decay of ^226^Ra away from the reservoir. ^210^Pb_unsup_ is deposited on particles of aerosols, supplied to a standing body of water, and along with the floating material, it is built into a sediment. Supported ^210^Pb_sup_ is formed in situ in a sediment through the decay of ^222^Rn, whose source is ^226^Ra which is contained in the sediment (Fig. 3). The sum of these two components is constituted by total lead. The estimated age was used to calculate the sedimentation rate of each layer (Szarlowicz et al. 2013; Zaborska et al. 2007).

## Results and discussion

Table 3 contains the ranges and means of the gamma radionuclide analyzed. All reported radioactivities are given in Becquerel per kilogram dry weight of sediments; the results were calculated for the date of sampling (supplementary materials). For both lakes, the ^40^ K radioactivities are fairly uniform along the sediment core. The mean ^40^ K radioactivities are different from the typical value of 1520 Bq∙kg^−1^ which characterizes the mean concentration in the Sucha Woda Gasienicowa Valley (Kubica et al. 2005). They are comparable to the mean value of ^40^ K (531 Bq∙kg^−1^) that represents the soils in Poland (CLOR 2021). The two sediment groups have different concentrations of ^226^Ra and ^228^Th. The radioactivity of ^226^Ra and ^228^Th in the individual layers of sediments taken from TSN is rather comparable (the levels are close to each other). This cannot be said for the second lake. The distribution of radionuclides, both ^226^Ra and ^228^Th, is rather chaotic without any dependence. ^226^Ra concentration is twice time higher than measured in soil collected from south Poland (CLOR 2021). In both lakes, the concentration of ^228^Th is a bit higher than ^228^Ra. It could be related to the composition of the mother rock and the mobility of these radionuclides. Radium in lakes can arise from natural sources such as groundwater inflow, sediment resuspension, resolubilization of sediment-bound radionuclides, and from air through precipitation and particle deposition. Thorium has generally been considered to be a lithophilic element of low geochemical mobility (IAEA 2014, Sheppard 1980).

**Table 3:** Radionuclides’ concentration [Bq∙kg^−1^]

Radionuclide	Toporowy Staw Niżni Lake				Toporowy Staw Wyżni Lake			
	Avg	Min	Max	sd	Avg	Min	Max	sd
^137^Cs	123	6.6	383	136	251	39.8	379	116
^40^ K	389	42.3	1019	241	528	3.7	1267	411
^228^Th	55.2	16.4	128	34.7	99.3	14.6	202	56.7
^226^Ra	86.4	30.4	197	40.3	84.7	9.3	204	52.4
^241^Am	5.0	1.3	13.2	4.4	4.9	0.3	7.3	2.8
^210^Pb_uns_	180	5.8	764	216	155	8.3	463	124

**Fig. 4:**
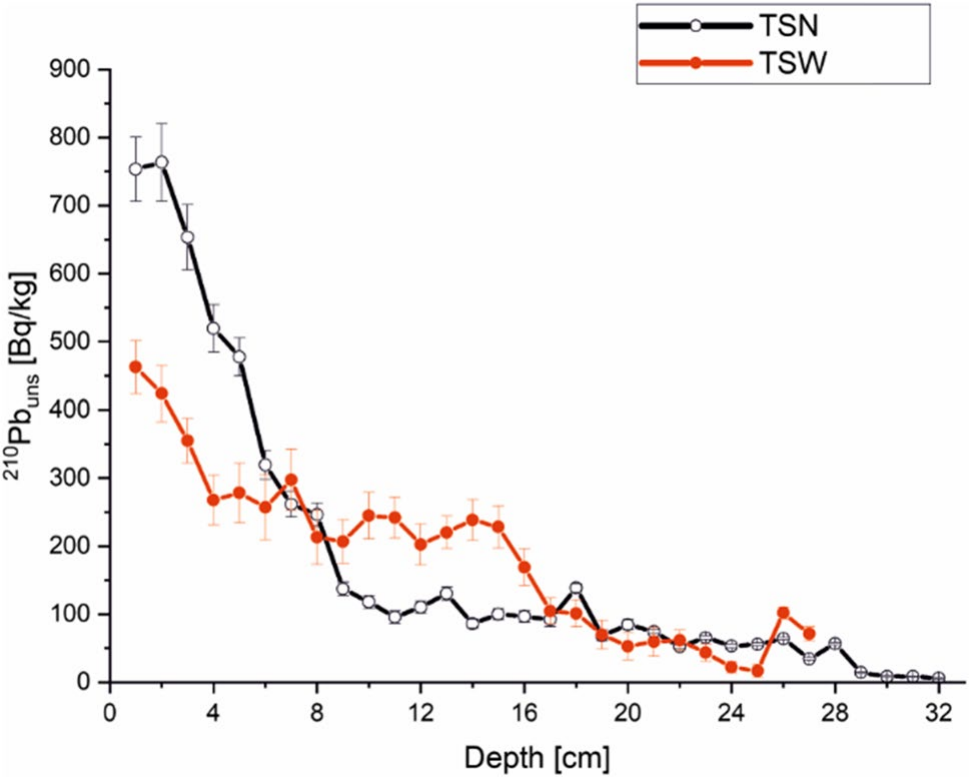
^210^Pb_uns_ distribution in sediment core from TSN and TSW

The value of unsupported ^210^Pb concentration was shown in Fig. 4, for both lakes. A nearly regular decrease in radioactivity of this radionuclide in the discussed sediment core was observed. In relation to the lead value, the obtained data can be interpreted in a study with the content of other sediments taken from the Tatra Mountains. The concentration of the lakes presented is 2 or 3 times lower. Comparable levels in the top layers of the profile can be found in Vysne Wahlenbergovo Pleso (2145 a.s.l). In relation to the Smreczyński Staw (1226 a.s.l.) which is also rich in organic matter, the ^210^Pb_uns_ content is slightly lower, approx. 200–100 Bq∙kg^−1^ (Appleby and Piliposian 2006).

**Fig. 5:**
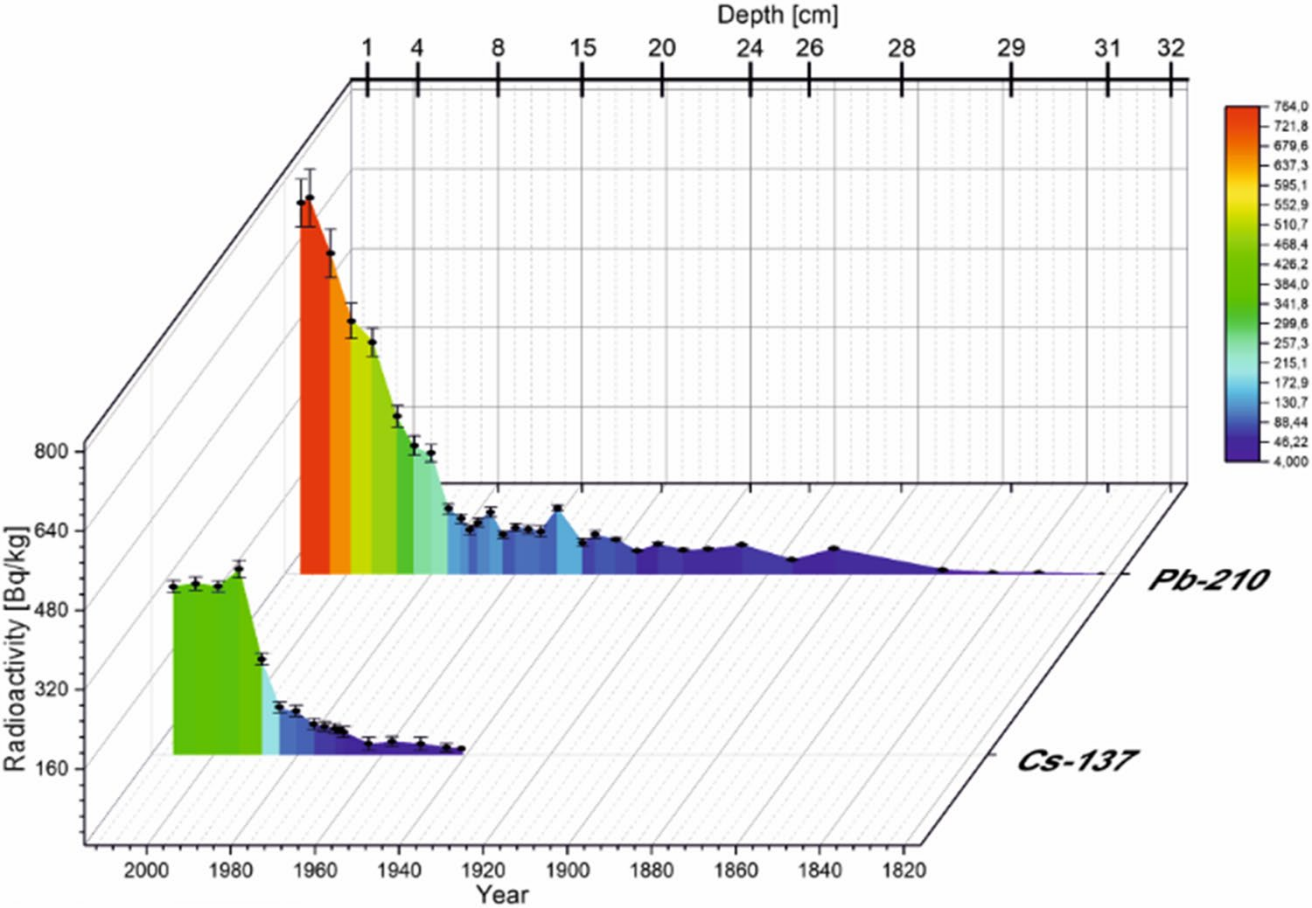
Depth-age model of the sediment based on ^210^Pb and ^137^Cs dating for TSN

**Fig. 6:**
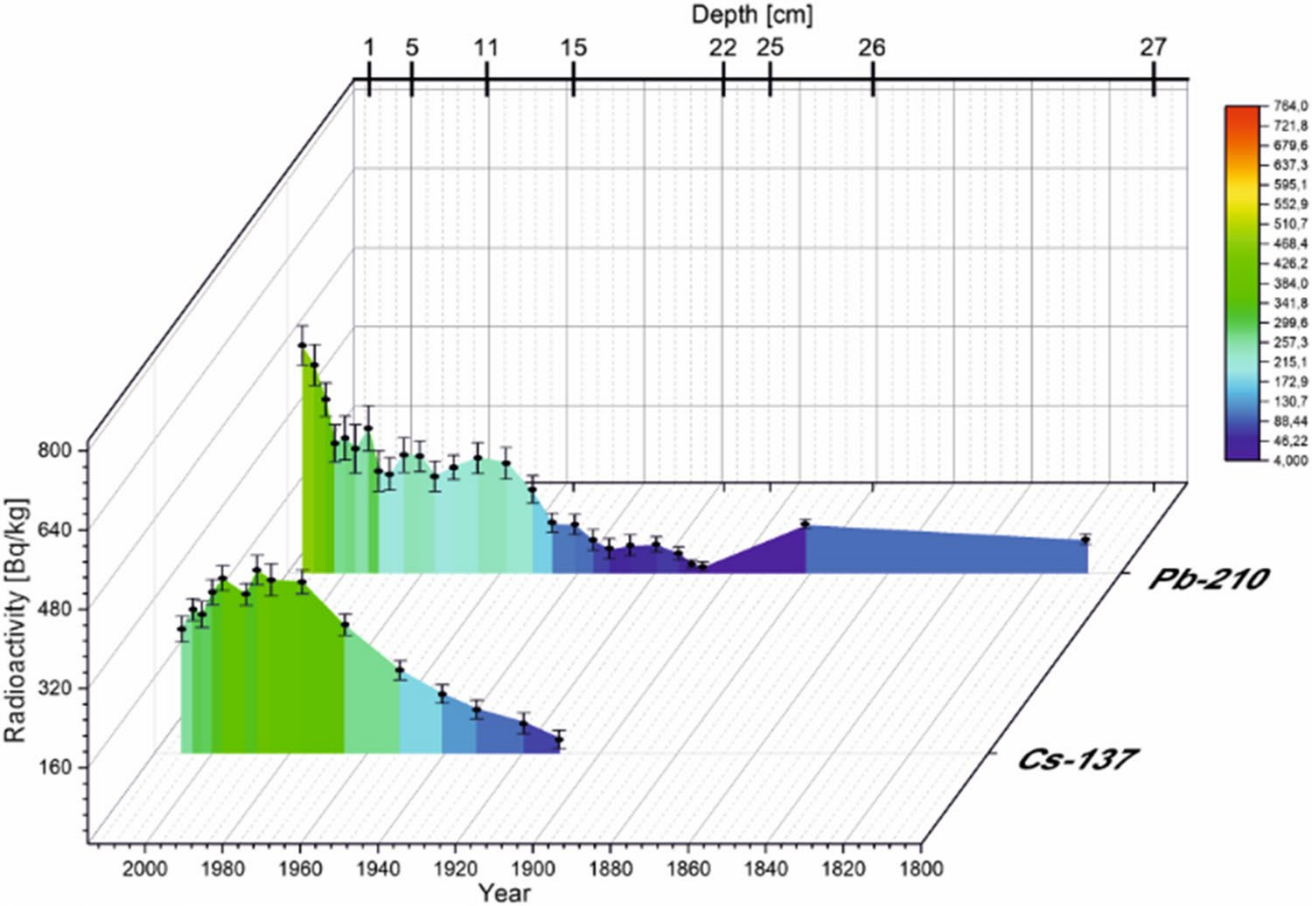
Depth-age model of the sediment based on ^210^Pb and ^137^Cs dating for TSW

Regarding ^137^Cs radioactivity, cesium radioactivity is at the highest level in the uppermost sediment layer and decreases with depth down the core (Figs. 5 and 6). In TSN, the level of ^137^Cs in the first four layers is comparable. A slight increase in the ^137^Cs radioactivity value occurs at 5-cm depth, which is the highest value of the concentration of this radionuclide in the entire sediment core. From 7- to 20-cm depth, the concentration of ^137^Cs is below 100 Bq∙kg^−1^. The same trend was observed for the Smreczyński Staw Lake, also a dystrophic lake with a similar geological structure. As for the ^137^Cs level, its amount is about 2 or even 3 times lower in TSN compared to Smreczyński Staw Lake. On the other hand, when comparing the ^137^Cs level in the TSW, between 2- and 16-cm depth, it remains at a level comparable to the top layers in the TSN. In both lakes, we have a downward trend toward the sediment core, which is related to the natural decay of this radionuclide and the lack of new supplies to the environment. The ^137^Cs presence in the deeper layers is related to its mobility in the sediments. This distribution is observed for lakes rich in organic matter (here 60–70%) (Szarlowicz and Kubica 2014). Americium was determined in the samples at the same depth as the maximum of ^137^Cs radioactivity or close to the layer which have high level of the ^137^Cs radioactivity.

CRS model with ^210^Pb dating was applicated with the aim of estimating the layer build-up time. In TSN the collected samples were dated back to 1820 (32 cm) (Fig. 5), and in TSW, the sediments composition comprises about 205 years (27 cm) (Fig. 6). The growth time of 1 cm of the layer in TSN is mostly between 2 and 5 years, and in the deepest layers, it reaches even 15 or even 26 years. The situation is slightly different in TSW for the first 9 cm; the creation time equals 3 years. From 10 to 25 cm, it is between 3 and 7 years with no regularity during sediment core. And the last dated layers were deposited for 26 or more years. Dates obtained from dating with ^210^Pb can usually be confirmed by the ^137^Cs distribution, because the time when it was delivered to the environment is perfectly known. Nevertheless, the situation here is difficult due to the ability of this radionuclide to move deeper into the sediment core. Generally, it is difficult to identify the maxima of the presence of ^137^Cs, but its increased concentration can be indicated. Regarding TSN the highest value of ^137^Cs was determined in the fifth layer, which corresponds to 1994. It is worth to add that ^241^Am can be also the time marker for the relevant sediment layer. Its elevated concentration is marked between 5 cm (13.2 Bq∙kg^−1^) and 6 cm (4.1 Bq∙kg^−1^) (1994–1988). The maximum fallout of ^137^Cs after testing with nuclear weapons, i.e., the 1960s, is not visible in the graph. Also the ^241^Am concentration was below lower limit of detection in the layer dedicated to 1950–1963 (18–14 cm). In TSW, at 9-cm depth (1988), the highest concentration of ^137^Cs occurs and decreases down the sediment core. In the TSW at 10-cm depth, an elevated concentration of ^241^Am (7.3 Bq∙kg^−1^) was observed, so both radionuclides can be considered as a time marker.

**Fig. 7:**
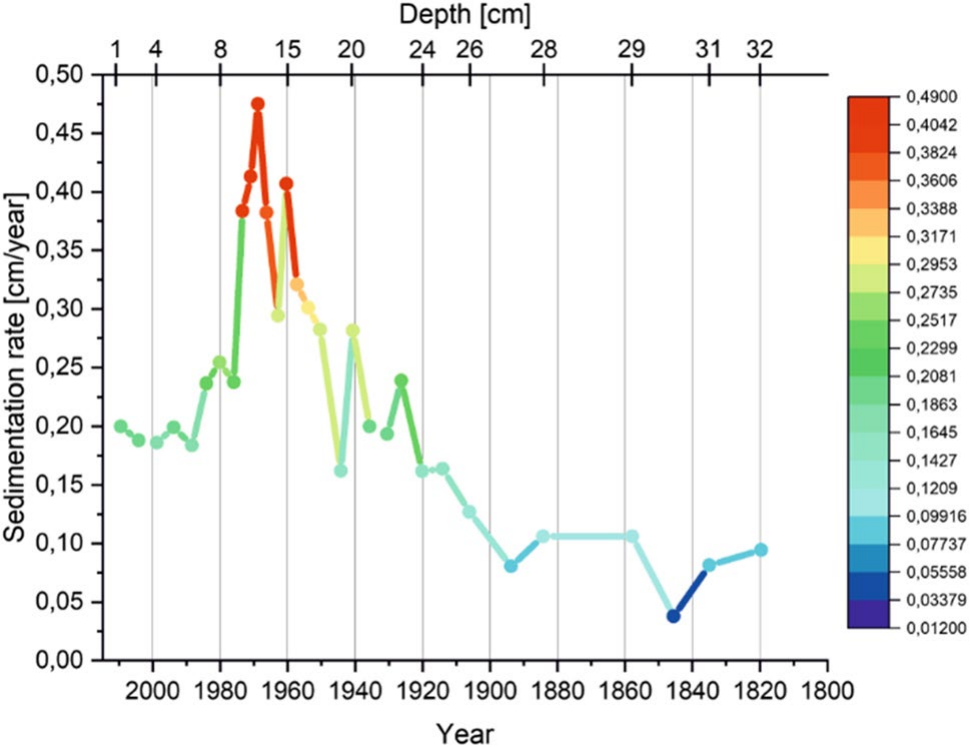
Changes of sedimentation rate in the studied period of time (TSN)

In TSN the change in sedimentation rate (Fig. 7) is irregular and ranges from 0.04 to 0.41 cm/year. In the lower layers of the sediment core, it shows much higher values than in the surface layers. This indicates an increased dynamics of material supply to the lake basin in the period of time between 1957 and 1973. However, in the deepest layers, the sedimentation rate shows the lowest value among the entire profile. Undoubtedly, due to the location of the lake, which is surrounded by slopes, the natural supply of material, whether in the form of dry or wet precipitation, surface runoff, and marginal erosion, has a huge impact. Changes in sedimentation rate are comparable to those observed in other mountain lakes (Appleby and Piliposian 2006). The highest level in the sedimentation rate values around 1960/1970 fits perfectly into the increased values of the amount of wet precipitation around 1970 (1627 mm/year) and 1962 (1599.8 mm/year, the highest value in recent years) (data gathered from the Institute of Meteorology and Water Management). It is also confirmed by the radioactivity of ^40^ K, ^228^Th, and ^226^Ra; around this period, they have increased value.

**Fig. 8:**
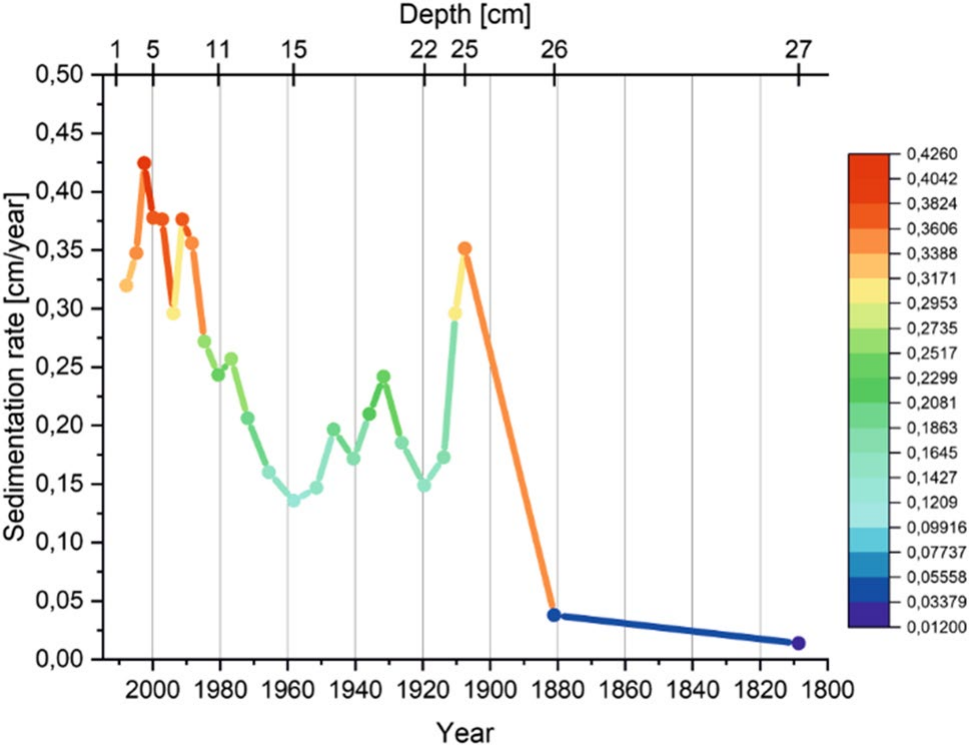
Changes of sedimentation rate in the studied period of time (TSW)

Taking into account the value of the sedimentation rate in TSW (Fig. 8), the variation was found to be between 0.01 and 0.42 cm/year. Higher values of ^210^Pb_uns_ in the first 11 cm of the sediment core correspond to a higher value of the sedimentation rate. In most layers, it took 3 years to deposit 1 cm of sediment. Between 4 and 12 cm, the level of ^210^Pb_uns_ shows comparable ranges, suggesting a similar or close source of supply, perhaps even the same. The entire course of changes in ^210^Pb concentrations may indicate that the lead present comes mainly from atmospheric precipitation (both dry and wet) and from the vicinity of the lake. Due to the flat surroundings of the lake (vegetation creating the surroundings in the form of peat-bog flora), the material is not supplied during surface runoffs, but not least because a large amount of material can be delivered to the lake during snow thaw or heavy rains.

Generally, the sedimentation process is very diverse and is individual for most lakes, depending on specific characteristics. Sometimes it is hard to explain the reason of changing the sedimentation rate, but the dynamic of sedimentological process could be related to few factors, e.g., water temperature conditions and the thermocline depth, vertical concentration gradients of dissolved or suspended particles, wind/wave influences and wave characteristics, sediment resuspension, and internal loading (Håkanson and Bryhn 2007). The original approaches of the mass balance model were presented by Vollenweider, which worked based on variables such as sedimentation, resuspension, and mixing (Vollenweider 1968). This model was developed by many authors, the most prominent work based on radiotracer analyst (Håkanson 2000). R.W. Fairbridge compilated these works and presented factors as follows: Sedimentation is the flux from water to sediments, resuspension from sediments back to water, concentration gradients in sediment to calculate diffusion from sediment, lake stratification, mineralization (bioactivity in sediment), solar irradiance (water bioactivity), inflow/outflow flux, and burial (Fairbridge et al. 2012).

**Fig. 9:**
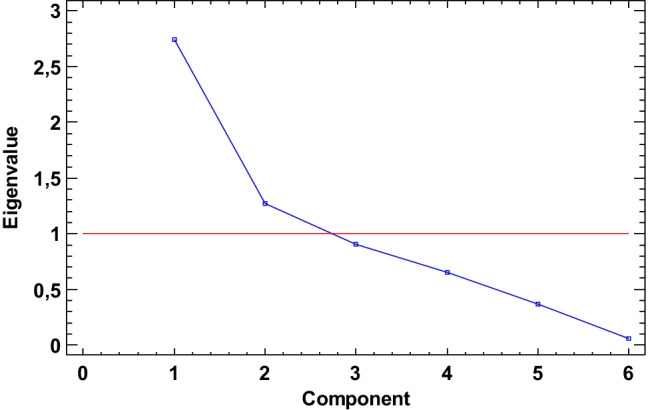
Scree plot

**Fig. 10:**
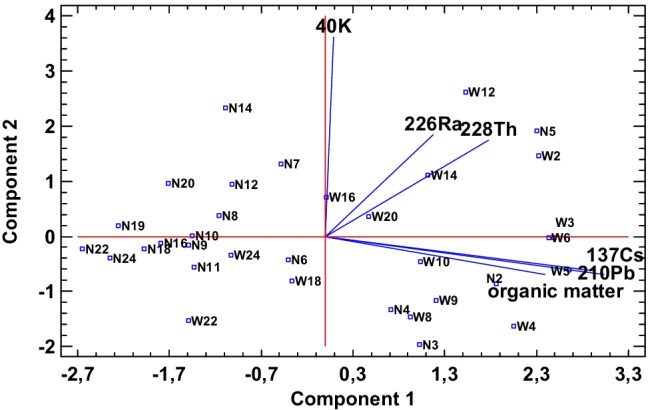
The biplot with projection of the variables and cases onto the plane for the Toporowe Stawy Lakes

Statistical data analysis (using Statgraphics Centurion 18) focused on data variables (^137^Cs, ^40^ K, ^228^Th, ^226^Ra, ^210^Pb, and organic matter). The number of complete cases is 33. Due to the fact that ^241^Am was determined only in few layers, it was not taken for statistical analysis. All results were standardized prior to chemometric analysis. Principal component analysis determines two main components. The Kaiser criterion and scree plot (Fig. 9) indicate that only two main components are necessary to data analysis. Table 4 presents the eigenvalue and percentage of variance. In Fig. 10, the biplot with the number of layers is shown. A positive correlation of ^137^Cs with ^210^Pb and organic matter was observed. ^137^Cs and ^210^Pb (mainly its unsupported part) have the same origin (taking into account the way it depositions in lakes). Their presence in the sediment is the result of an external supply from outside the lake. The ^40^ K content is orthogonal to all other variables, which proves a complete lack of ^40^ K correlation with other determined radioisotopes. ^226^Ra and ^228^Th are partially correlated, which is justified by the fact that the mineral part of the sediments comes from the same parent rock, in which the ratio of their concentrations is constant. The differences are only due to the different mobility (depending on the changing pH) of ^226^Ra and ^228^Th in the water environment. The biplot shows the division between the sediments collected from the TSW and TSN. The majority of sediments from TSN are focused on the left site of the biplot and the sediments from TSW on the right site. Thanks to the application of principal component analysis (PCA), it was possible to demonstrate that ^137^Cs, ^210^Pb, and organic matter are the main differentiating significance of the examined lakes. The same is assigned to ^226^Ra and ^228^Th but to a lesser extent (probably due to the proximity of the lakes), and ^40^ K, as mentioned, practically does not differentiate the tested samples.

**Table 4:** Principal component analysis

Component		Percent of variance	Cumulative percentage
Number	Eigenvalue		
1	2.744	45.747	45.747
2	1.269	21.155	66.902
3	0.902	15.033	81.934
4	0.653	10.881	92.816
5	0.369	6.144	98.960
6	0.062	1.040	100.000

**Fig. 11:**
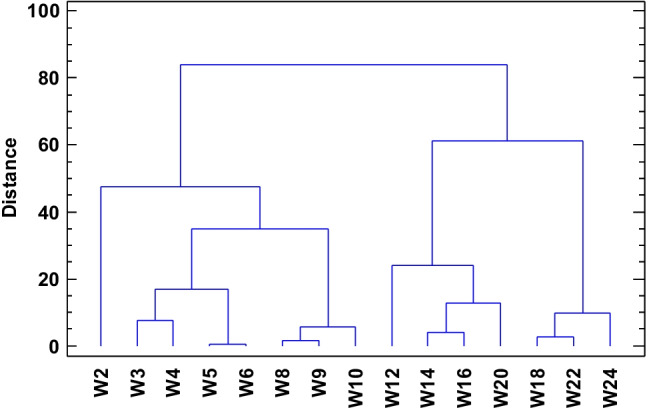
Cluster analysis for the TSW Lake. Cases agglomerated with Ward’s method, Euclidean distance

**Fig. 12:**
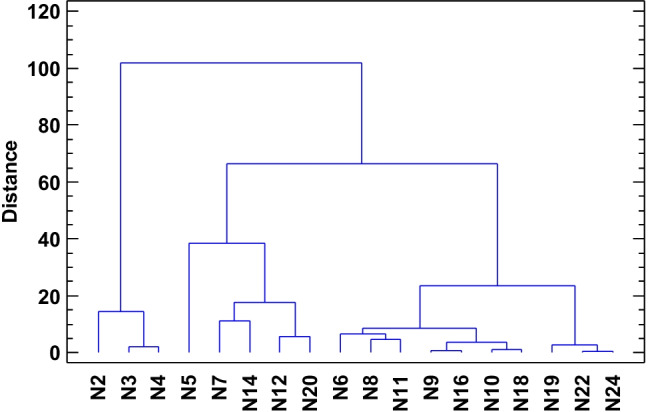
Cluster analysis for the TSN Lake. Cases agglomerated with Ward’s method, Euclidean distance

To compare the layers, the graphical presentation of cluster analysis with Ward’s method and Euclidean distance was done separately for each reservoir. Some of the clusters are relatively easy to explain which relates to grouping the layers showing similarities (Figs. 11 and 12). The similarities between the layers can be interpreted in relation to the obtained values of sedimentation rates. Most of the layers in the range up to W10 are in the first main cluster distinguished with an emphasis on similarities between some of them. Looking at the W5 i W6 as well as W8 and W9 layers, they have very similar sedimentation rate values among themselves, but the differences between them resulted in the identification of separate subclusters. The second major cluster includes the W12 to W24 layers. For example, there are also subclusters, e.g., W14 and W16 and W18 and W22 showing similarity, which can also be associated with similar value of sedimentation rate within their cluster. The situation is different in TSN (Fig. 12). The division into two main groups was also distinguished. Nevertheless, clearly specified in one of them, covering the layers from N5, two subclusters are further developed into smaller ones. It is difficult to identify the reason for determining the division of layers. The analysis of similarities showed that the outermost layers (N2, N3, N4) are similar and, what is more, they differ from the others so much that they created a separate cluster in the analysis.

## Conclusions

In summary, sediment is a valuable source of information about the condition and quality of the environment of a given aquatic ecosystem. It should be emphasized that the amount of sediment collected in sediment core from mountain reservoirs is small compared to global analyses of sediments from other aquatic ecosystems. The limitation in sediment collection from mountain lakes is the fact that it is located in a protected area, the lake route, or the selection of samplers. However, to determine the time of formation of a given sediment layer as accurately as possible, it is advisable to collect the material of the smallest possible thickness, which has a negative impact on the amount of material for analysis. Undoubtedly, the method of determining unsupported lead using its daughter radionuclide (^210^Po) is one of the most sensitive measurement methods. Therefore, a detailed analysis of environmental changes on a time scale and a quantitative determination of the sedimentation rate can be done through the ^210^Pb radionuclide. An important value is the determination of the content of other radionuclides in the collected layers, which was a huge analytical challenge for gamma radiation spectrometry because of the small portion of the material.

Based on the research, the following could be concluded:

1. Location, surroundings, and low water retention affect the level and distribution of radionuclides in both lakes. The TSW lake is characterized by slightly higher mean concentrations of radionuclides due to the smaller lake area, catchment area, and water table as well as lower retention, which causes more frequent concentration and precipitation from the lake waters.
2. Based on the distribution of radionuclides, there is the possibility to estimate the sources/origin of material supply to the catchment area, such as and factors that influence the sedimentation process.
3. Despite the difficulties in interpreting the measurement maxima dedicated to ^137^Cs, its applicability as a time marker was confirmed thanks to the presence of ^241^Am.
4. The measured radionuclides in the sediment core, on the one hand, give the level of concentration of the radionuclide of a given area and, on the other hand, are a tool for the interpretation of changes that take place in the aquatic environment.
5. The basic statistical tools applied confirmed the sources of radionuclide supply to the lake basin and showed the similarities between the profile layers.
6. The PCA analysis allowed for the reduction of the number of variables, which simplified the interpretation of the variability of radionuclide content in the sediments and showed the diversity of the examined objects.

The submitted analyses contribute new valuable material to the knowledge regarding the analysis of environmental changes based on radioisotope studies of sediments from mountain areas. It has been proven that small water reservoirs, situated in the lower parts of the mountains, can also have great potential. The research results obtained can be used as comparative material for other alpine lakes, in particular lakes with no outflow, which are rich in organic matter. The entire analysis carried out allows us to assume that as long as the overgrown lake has a water hole with an appropriate area and does not dry up during the year, the sediments collected from such a water ecosystem will constitute a rich scientific potential, and the applied geochronological dating based on ^210^Pb will be a valuable tool in interpretation of changes that occur therein.

## Supplementary Information

Below is the link to the electronic supplementary material.

Supplementary file1 (DOCX 24 KB)

## Data Availability

All data analyzed during this study are included in this published article and are not publicly available but may be obtained from the corresponding author on reasonable request.
