# Supplementary material for: Sedimentary conditions based on the vertical distribution of radionuclides in small dystrophic lakes: a case study of Toporowe Stawy Lakes (Tatra Mountains, Poland)
Source: Environ Sci Pollut Res Int. 2022 Jul 19;29(59):89530–41. doi: 10.1007/s11356-022-21922-3 (PMC9672015; doi:10.1007/s11356-022-21922-3)
Supplement: Supplementary file 1 — Supplementary file1 (DOCX 24 KB) [file 11356_2022_21922_MOESM1_ESM.docx]

Supplementary materials

TSN Lake

| **Depth [cm]** | **Radioactivity Bq·kg^-1^ ^*^** | | | | |
| --- | --- | --- | --- | --- | --- |
|  | ^137^Cs | ^241^Am | ^40^K | ^228^Th | ^226^Ra |
| 2 | 348 | LLD | 269 | 41.4 | 106 |
| 3 | 353 | 5.6 | 42.3 | 37.2 | 33.3 |
| 4 | 348 | 4.4 | 109 | 54.8 | 55.0 |
| 5 | 383 | 13.2 | 740 | 128 | 197 |
| 6 | 194 | 4.1 | 232 | 40.5 | 97.1 |
| 7 | 100.9 | LLD | 535 | 102 | 138 |
| 8 | 93.7 | LLD | 343 | 53.0 | 119 |
| 9 | 68.0 | ≈1.3 | 248 | 27.4 | 104 |
| 10 | 63.3 | LLD | 351 | 51.2 | 74.7 |
| 11 | 59.0 | LLD | 72.9 | 46.3 | 90.6 |
| 12 | 52.8 | LLD | 566 | 108 | 75.4 |
| 14 | 29.8 | LLD | 1019 | 103 | 106 |
| 16 | 34.3 | LLD | 360 | 16.4 | 82.7 |
| 18 | 29.4 | LLD | 396 | 25.8 | 50.7 |
| 19 | 21.7 | LLD | 414 | 24.0 | 79.9 |
| 20 | 20.2 | ≈1.4 | 602 | 88.3 | 65.0 |
| 22 | 14.0 | LLD | 405 | 24.1 | 30.4 |
| 24 | 6.6 | LLD | 296 | 22.9 | 49.4 |

^*^uncertainty for ^137^Cs is below 10%, for other is between 15%-40%

TSW Lake

| **Depth [cm]** | **Radioactivity Bq·kg^-1^ ^*^** | | | | |
| --- | --- | --- | --- | --- | --- |
|  | ^137^Cs | ^241^Am | ^40^K | ^228^Th | ^226^Ra |
| 2 | 262 | LLD | 989 | 14.6 | 204 |
| 3 | 300 | LLD | 667 | 108 | 54.2 |
| 4 | 291 | LLD | LLD | 145 | 9.3 |
| 5 | 335 | LLD | LLD | 150 | 118 |
| 6 | 362 | 6.4 | 260 | 129 | 126 |
| 8 | 330 | 6.6 | LLD | 67.5 | 59.3 |
| 9 | 379 | LLD | LLD | 35.5 | 126 |
| 10 | 359 | 7.3 | LLD | 101 | 130 |
| 12 | 355 | LLD | 1267 | 202 | 50.2 |
| 14 | 267 | LLD | 602 | 163 | 88.5 |
| 16 | 177 | 4.1 | 755 | 91.9 | 50.1 |
| 18 | 130 | LLD | 125 | 73.2 | 57.7 |
| 20 | 99.3 | LLD | 114 | 149 | 128 |
| 22 | 71.6 | ≈0.3 | ≈3.7 | 37.8 | 20.8 |
| 24 | 39.8 | LLD | 495 | 23.7 | 49.1 |

^*^uncertainty for ^137^Cs is below 10%, for other is between 15%-40%
